# Oncogenic drivers and mitochondrial dependency

**DOI:** 10.18632/aging.100734

**Published:** 2015-03-26

**Authors:** Francesca Alvarez-Calderon, James DeGregori

**Affiliations:** ^1^ Integrated Department of Immunology, Medical Scientist Training Program, School of Medicine, University of Colorado Anschutz Medical Campus, Aurora, CO 80045; ^2^ Department of Biochemistry and Molecular Genetics; ^3^ Department of Pediatrics, Division of Hematology, Oncology and Bone Marrow Transplantation; ^4^ Department of Medicine, Section of Hematology, University of Colorado Anschutz Medical Campus, Aurora, CO 80045

Most metabolically targeted approaches to cancer therapy have focused on the reliance of cancer cells on glycolysis for energy metabolism. The Warburg effect is thought to minimize the role of mitochondrial metabolism in the survival of cancer cells. We recently demonstrated that BCR-ABL-driven leukemic cells become reliant on the TCA cycle for energy production upon inhibition of the dominant tyrosine kinase (TK) [1]. While normally relatively dispensable in these leukemia cells, carbon entry into the mitochondria via pyruvate dehydrogenase becomes critical for energy homeostasis and survival following TK inhibition. Similarly, Zhao et al. demonstrated that HIF1α-mediated acquired resistance to TK inhibition in BCR-ABL-dependent leukemias engenders enhanced sensitivity to oxythiamine, an inhibitor of both the pyruvate dehydrogenase complex and the non-oxidative pentose phosphate pathway enzyme transketolase [2]. Furthermore, inhibition of the driving oncogenic kinase, BRAF, in melanoma led to increased expression of TCA cycle enzymes as well as oxidative phosphorylation and ATP synthesis genes, revealing a previously unappreciated mechanism of survival in these cells via increased flux through the TCA cycle [3].

In addition, Kluza et al. profiled the activity of enzymes in the electron transport chain (ETC) in human and mouse BCR-ABL cell lines harboring resistance to the BCR-ABL kinase inhibitor imatinib mesylate (IM) and found that IM-resistant cells had a reduction in Complex I, II and IV activity, which correlated with the protein expression of the different components [4]. This resistance came with a cost – increased reactive oxygen species (ROS) levels and heightened sensitivity to pro-oxidants. Inhibition of glycolysis in these IM-resistant leukemias leads to derepression of mitochondrial respiration, increased flux through the TCA cycle, and reduced levels of ROS [4]. Thus, either inhibition of a driving TK or the development of TK resistance can alter the dependence of leukemia cells on mitochondrial carbon use, engendering new metabolic vulnerabilities.

Furthermore, we have demonstrated that the altered mitochondrial dependencies following inhibition of the dominant TK extend beyond mitochondrial carbon flux and respiration. Inhibition of the driving TK in leukemia cells (using IM or dasatinib in BCR-ABL-driven leukemias, quizartinib in FLT3-driven leukemia, and IM in KIT-driven leukemia) makes these cells exquisitely sensitive to low doses of oligomycin-A, an inhibitor of mitochondrial ATP synthase, highlighting particular druggable dependencies in leukemic cells that are exposed to TK inhibition [1]. Interestingly, these low nmol/L doses of oligomycin-A do not inhibit oxygen consumption, a readout of ETC function, but rather lead to transient decreases in ATP levels and changes in mitochondrial membrane potential (Ψ_m_). Interestingly, three additional groups have used large-scale proteomic and/or transcriptomic analyses to identify pathways that are altered upon inhibition of a dominant oncogene. They identified subpopulations of tumor cells in melanoma and pancreatic adeno-carcinoma that, upon treatment with cytotoxic drugs, inhibition of the driving oncogene or withdrawal of the dominant oncogene, upregulate components involved in the ETC and oxidative phosphorylation [3, 5, 6]. Two of these groups used low doses of oligomycin-A, in combination with cisplatin or KRAS-withdrawal, to target surviving cancer cells, leading to long-term reductions in clonogenic activity. While these authors concluded that oncogene inhibition restored dependence on mitochondrial respiration, the doses of oligomycin-A that showed efficacy are below those capable of inhibiting respiration. That inhibition of mitochondrial respiration is not required for the anti-cancer efficacy of oligomycin-A may underlie its effectiveness and lack of toxicity in mouse models [1], and furthermore, may allow its utilization in humans. In essence, inhibition of the driving oncogene in some cancers appears to generate a therapeutic window for oligomycin-A mediated impairment of some mitochondrial function.

In pancreatic adenocarcinoma cells, ablation of KRAS caused hyperpolarization of the mitochondrial membrane and increased ROS production [6]. This phenotype is consistent with increased supply of electron donors to the ETC, leading to increased generation of ROS. Our study shows that the ability of oligomycin-A to synergize with TK inhibition in the elimination of leukemia cells relies on the TK-mediated inhibition of glycolysis, is partially dependent on the generation of ROS, and coincides with reduced Ψ_m_ [1]. Thus, while inhibition of mitochondrial respiration does not appear to underlie the anti-cancer potential of oligomycin-A, some yet to be defined mitochondrial function appears to be key.

These studies highlight a potential ubiquitous vulnerability of tumor cells that survive both targeted and genotoxic therapies, sensitivity to mitochondrial perturbations, that could provide a “second hit” to target quiescent or dormant cancer cells. The biochemical nature of this vulnerability needs to be more fully defined. Nonetheless, it could provide the lethal blow to cancers for which targeted therapies have proven insufficient to eliminate the malignancy and in slow-cycling cancer cell subpopulations that are inherently resistant to genotoxic and radiation-based therapies geared towards rapidly dividing cells [5].
